# Editorial Comment: Impact of Obesity on Perioperative Outcomes at Robotic-assisted and Open Radical Prostatectomy: Results From the National Inpatient Sample

**DOI:** 10.1590/S1677-5538.IBJU.2021.01.07

**Published:** 2020-11-18

**Authors:** Eliney F. Faria

**Affiliations:** 1 Hospital Felicio Rocho Departamento de Urooncologia e Cirurgia Robotica Belo Horizonte Brazil Departamento de Urooncologia e Cirurgia Robotica, Hospital Felicio Rocho - Belo Horizonte

Knipper S ^1^, Mazzone E ^2^, Mistretta FA ^3^, Palumbo C ^4^, Tian Z ^5^, Briganti A ^6^

## COMMENT

Obesity is a growing public health issue worldwide and in this paper Dr. Sophie Knipper and cols. emphasized that regardless of the surgical technique, open or robotic-assisted, obese patients (BMI ≥30 kg/m2) may be predisposed to more frequent adverse perioperative outcomes (1). They included for their analyses a control-group of nonobese patients and accessed the National Inpatient Sample (NIS) database from 2008 to 2015 (2), meaning 20% of United States inpatient hospitalizations. They used the World Health Organization (WHO) definition for obese patients. In a very good statistical analysis they found interesting data (3). Of all 89,383 underwent to radical prostatectomy, 7.9% were obese. Overall complications were recorded in 13.1 vs 7.9% of obese vs nonobese robotic-assisted radical prostatectomy (RARP) and 17.4 vs 11.3% of obese vs nonobese underwent to open radical prostatectomy (ORP) (both p < 0.001). Medical complications were recorded in 7.7 vs 4.4% of obese vs nonobese RARP and in 8.3 vs 5.6% of obese vs nonobese ORP (both p < 0.001). Cardiac, respiratory and genitourinary complications had higher rates in obese vs nonobese patients (all p < 0.001). Obese patients had more days of hospital staying and more costs (both p < 0.001). However the multivariable analyses showed RARP had fewer rates of complications than ORP in obese patients. They conclude that obese patients are predisposed to higher rates of adverse peri-and postoperative outcomes. The authors addressed that potentially more favorable outcomes are observed in obese patients when RARP is used instead of ORP. They concluded despite higher costs of RARP in comparison with ORP the obese patients had benefits with this technology.
